# Autosomal dominant non-syndromic hearing loss maps to *DFNA33* (13q34) and co-segregates with splice and frameshift variants in *ATP11A,* a phospholipid flippase gene

**DOI:** 10.1007/s00439-022-02444-x

**Published:** 2022-03-12

**Authors:** Justin A. Pater, Cindy Penney, Darren D. O’Rielly, Anne Griffin, Lara Kamal, Zippora Brownstein, Barbara Vona, Chana Vinkler, Mordechai Shohat, Ortal Barel, Curtis R. French, Sushma Singh, Salem Werdyani, Taylor Burt, Nelly Abdelfatah, Jim Houston, Lance P. Doucette, Jessica Squires, Fabian Glaser, Nicole M. Roslin, Daniel Vincent, Pascale Marquis, Geoffrey Woodland, Touati Benoukraf, Alexia Hawkey-Noble, Karen B. Avraham, Susan G. Stanton, Terry-Lynn Young

**Affiliations:** 1 Faculty of Medicine, Memorial University, 300 Prince Phillip Drive, St. John’s, NL Canada; 2 Dana-Farber Cancer Institute, Harvard Medical School, Boston, MA USA; 3 Centre for Translational Genomics, Memorial University, 300 Prince Phillip Dr., St. John’s, NL Canada; 4 Department of Human Molecular Genetics and Biochemistry, Faculty of Medicine and Sagol School of Neuroscience, Tel Aviv University, 6997801 Tel Aviv, Israel; 5 Institute of Human Genetics, University Medical Center Göttingen, Göttingen, Germany; 6 Institute for Auditory Neuroscience and InnerEarLab, University Medical Center Göttingen, Göttingen, Germany; 7 Institute of Medical Genetics, Wolfson Medical Center, 58100 Holon, Israel; 8 Bioinformatic Center, Cancer Research Institute, The Wohl Institute for Translational Medicine, Sheba Medical Center, Tel-Hashomer, Israel; 9 Sackler School of Medicine, Tel Aviv University, Tel Aviv, Israel; 10 Communication Sciences and Disorders, Elborn College, Western University, 1201 Western Road, London, ON Canada; 11 The Lorry I. Lokey Center for Life Sciences and Engineering, Technion-Israel Institute of Technology, Haifa, Israel; 12 The Centre for Applied Genomics, The Hospital for Sick Children, Peter Gilgan Centre for Research and Learning, 686 Bay Street, Toronto, ON Canada; 13 Genome Quebec Innovation Centre, McGill University, 740 Dr. Penfield Avenue, Montreal, QC Canada; 14 Canadian Centre for Computational Genomics, McGill University and Genome Quebec Innovation Center, 740 Dr. Penfield Avenue, Montreal, QC Canada

## Abstract

Sequencing exomes/genomes have been successful for identifying recessive genes; however, discovery of dominant genes including deafness genes (DFNA) remains challenging. We report a new DFNA gene, *ATP11A*, in a Newfoundland family with a variable form of bilateral sensorineural hearing loss (SNHL). Genome-wide SNP genotyping linked SNHL to *DFNA33* (LOD = 4.77), a locus on 13q34 previously mapped in a German family with variable SNHL. Whole-genome sequencing identified 51 unremarkable positional variants on 13q34*.* Continuous clinical ascertainment identified several key recombination events and reduced the disease interval to 769 kb, excluding all but one variant. *ATP11A* (NC_000013.11: chr13:113534963G>A) is a novel variant predicted to be a cryptic donor splice site. RNA studies verified in silico predictions, revealing the retention of 153 bp of intron in the 3′ UTR of several *ATP11A* isoforms. Two unresolved families from Israel were subsequently identified with a similar, variable form of SNHL and a novel duplication (NM_032189.3:c.3322_3327+2dupGTCCAGGT) in exon 28 of *ATP11A* extended exon 28 by 8 bp, leading to a frameshift and premature stop codon (p.Asn1110Valfs43Ter). *ATP11A* is a type of P4-ATPase that transports (flip) phospholipids from the outer to inner leaflet of cell membranes to maintain asymmetry. Haploinsufficiency of ATP11A, the phospholipid flippase that specially transports phosphatidylserine (PS) and phosphatidylethanolamine (PE), could leave cells with PS/PE at the extracellular side vulnerable to phagocytic degradation. Given that surface PS can be pharmaceutically targeted, hearing loss due to *ATP11A* could potentially be treated. It is also likely that *ATP11A* is the gene underlying *DFNA33*.

**Supplementary Information:**

The online version contains supplementary material available at 10.1007/s00439-022-02444-x.

## Introduction

Hereditary hearing loss is a common sensory disorder exhibiting extensive genetic and clinical heterogeneity (Morton and Nance 2006). Over 200 hearing loss genes have been identified (Van Camp and Smith 2015); however, approximately one-third of the 60 mapped autosomal dominant loci (*DFNA*) evade discovery. Like other autosomal dominant traits, hearing loss is typically characterized by variable expressivity and reduced penetrance (Richard et al. 1999). Whole-genome sequencing (WGS) in patients with dominant conditions uncovers many heterozygous variants, which is particularly problematic when the underlying gene is novel. Well-ascertained multiplex families and more traditional methods (e.g., linkage and haplotype analysis) can be critical to reduce the number of candidate variants co-segregating with the trait of interest. Well-ascertained families also provide truly unaffected members that can be used to filter out benign sequencing variants. Genetic isolates can also help where apparently unrelated families are members of a clan (Sherry et al. 2001), as well as the fact that sibships are usually large and genealogical connections with distant relatives known in close knit communities. With the increased power to detect rare variants using genome compared with exome sequencing, genome analysis should include all known isoforms of candidate genes, and not be limited to validated isoforms (i.e., RefSeq) (Belkadi et al. 2015; Zhao and Zhang 2015).

A key feature of eukaryotic cell membranes is the non-random (asymmetrical) distribution of phospholipids, an essential feature to maintaining cell integrity (Segawa et al. 2014). Asymmetry is most evident at the plasma membrane (Zachowski 1993) and is maintained by the action of three classes of proteins: scramblases, floppases and flippases. Phospholipid flippases (or P4-ATPases) specifically transport or “flip” phospholipids from the outer to the inner leaflet (Paulusma and Elferink 2010). Mouse studies have demonstrated the importance of the P4-ATPase class of flippases in phospholipid metabolism and maintaining normal auditory function (Coleman et al. 2014; Stapelbroek et al. 2009). For example, pathogenic variants in *ATP8B1* (MIM: 605868) cause intrahepatic cholestasis type 1 (MIM: 211600), where patients sporadically develop hearing loss (Stapelbroek et al. 2009). Phospholipids have also been implicated in autoimmune conditions, such as antiphospholipid syndrome characterized by the presence of antiphospholipid antibodies that invoke an autoimmune response causing thrombosis, and complications during pregnancy and hearing loss (Mouadeb and Ruckenstein 2005; Wiles et al. 2006).

Herein, we map variable sensorineural hearing loss (SNHL) in a family of Northern European descent to the *DFNA33* locus (OMIM 614211) who presented a novel likely pathogenic splice variant in the 3′ region of the *ATP11A* gene (OMIM: 605868). Subsequently, we identified a pathogenic duplication variant in two unrelated Jewish Israeli families with variable SNHL*.*

## Materials and methods

**Fig. 1:**
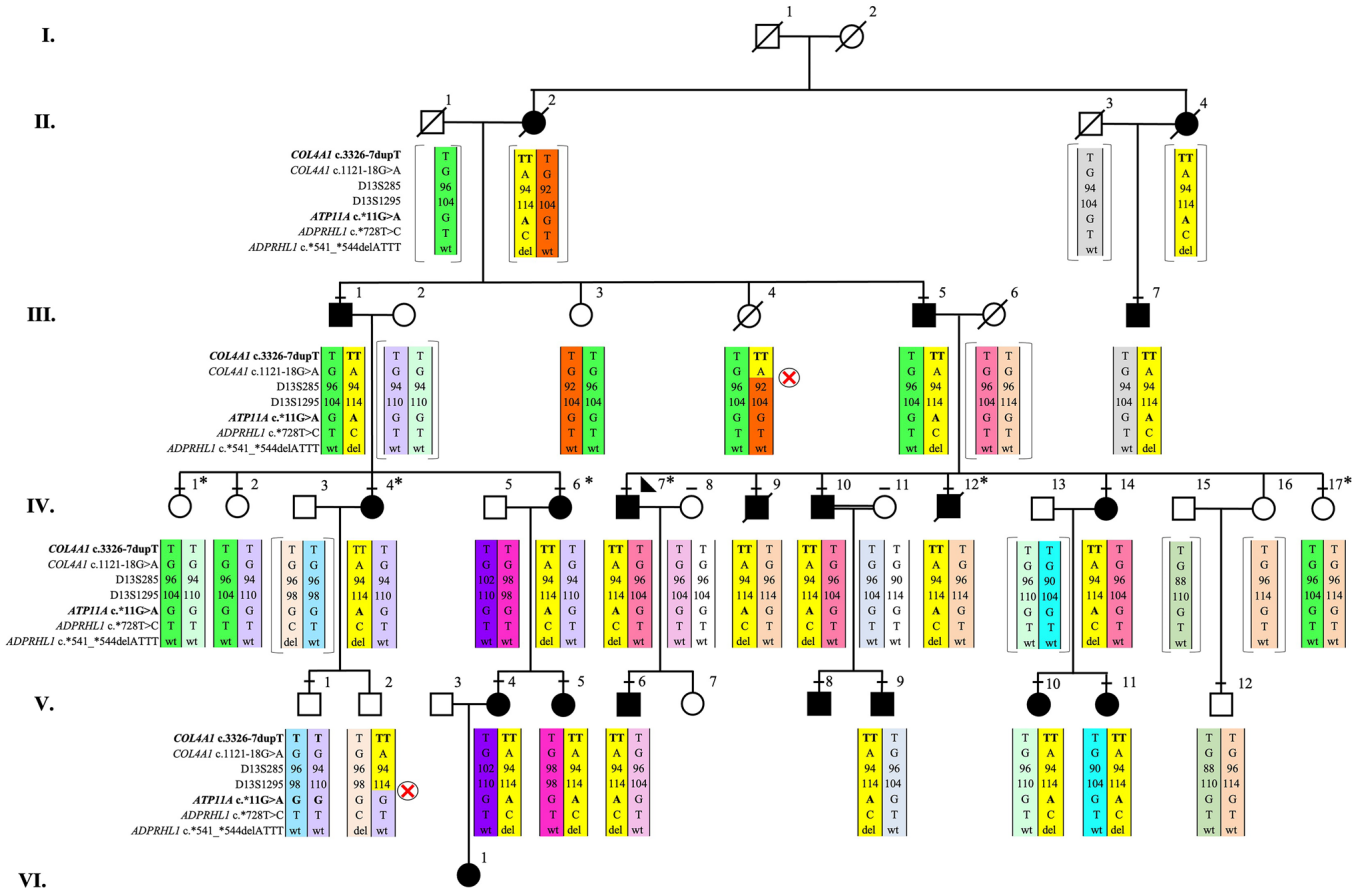
Six generation Newfoundland pedigree with autosomal dominant bilateral SNHL segregating a *DFNA33*-linked disease haplotype on 13q34. Haplotypes (colored vertical bars) represent a 3.6 Mb region on chromosome 13q34 with polymorphic markers and SNPs and their relative genomic positions (left). The disease haplotype (yellow) is diminished by several key crossover events (red x). Squares and circles represent males and females, respectively. Dashes above symbols indicate pure tone audiometry hearing testing was performed. Asterisks are the members that were selected for genome sequencing. Shaded symbols denote hearing loss

**Fig. 2:**
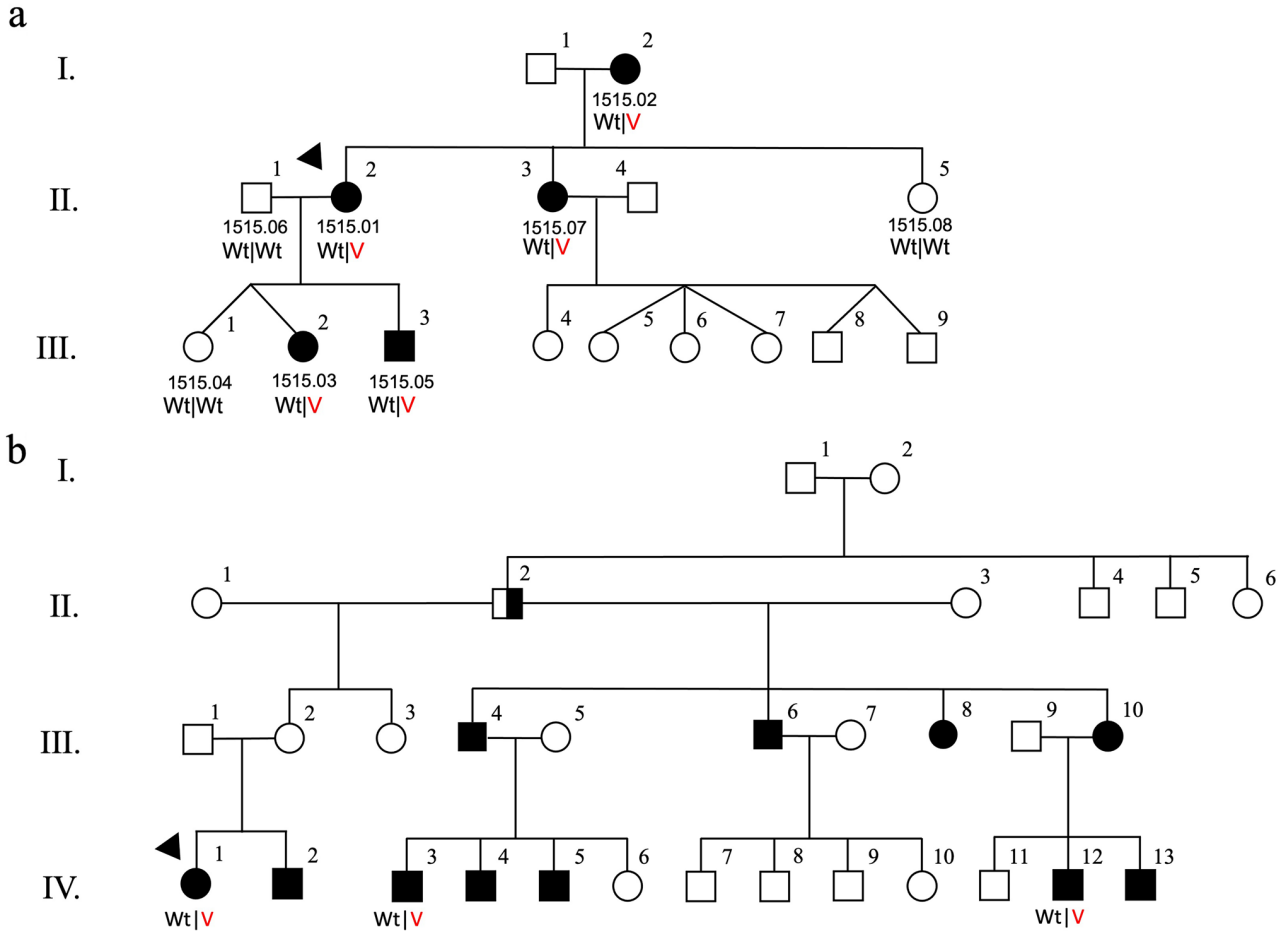
Recurrent *ATP11A* variant shared between two Jewish Israeli families with bilateral sensorineural hearing loss as an autosomal dominant trait. Hearing loss and a novel duplication in exon 28 of *ATP11A* (NM_032189.3:c.3322_3327+2dupGTCCAGGT) that occurs within the splice donor sequence (intron 28) segregate across three generations in Family A (**a**). The proband (PID IV-1) from Family B (**b**) presented for clinical exome sequencing that was completed on her and her two cousins (PIDs IV-3 and IV-12). All screened negative for DFNA genes but were heterozygous for a novel duplication in exon 28 of *ATP11A* (NM_032189.3:c.3322_3327+2dupGTCCAGGT) that occurs within the splice donor sequence (intron 28). Squares and circles represent males and females, respectively. Half and full shaded symbols denote severe congenital and moderate hearing loss, respectively. Wt: wild-type allele, V: *ATP11A* c.3322_3327+2dupGTCCAGGT

**Fig. 3:**
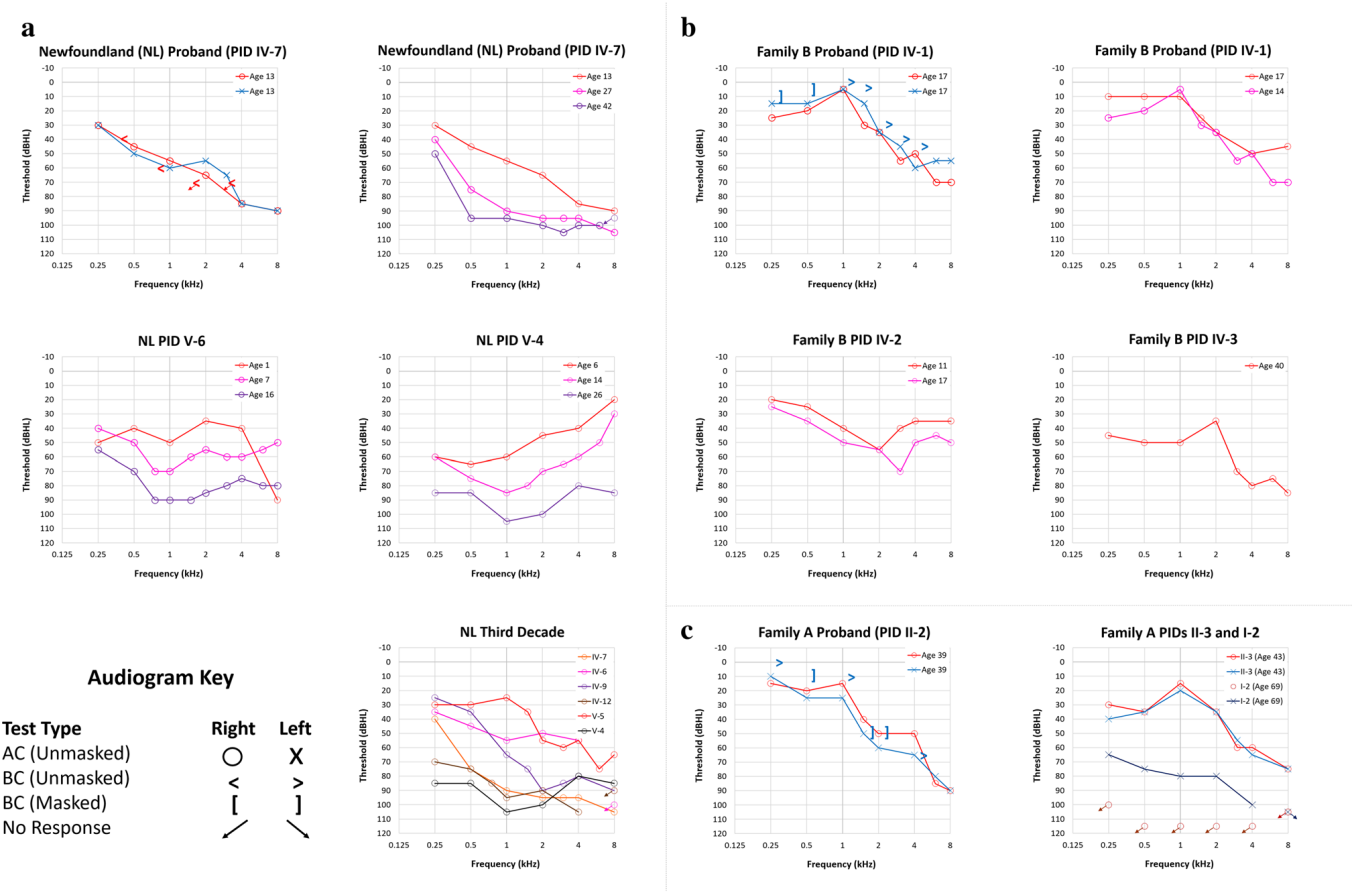
Audiograms of three families with autosomal dominant, variable non-syndromic hearing loss and novel variants in the *ATP11A* gene. **a** Audiograms of the Newfoundland (NL) family with the *ATP11A* (NC_000013.11:chr13:113534963G>A) variant. Full audiogram of the proband (PID IV-7; upper left). Audiograms (right ear series) of the proband and two family members show variable configuration and progression of hearing loss (top right and middle). Comparison of audiograms (right ear series) of six family members show variable severity in the 3rd decade (PIDs IV-7 age 27 years; IV-6 age 28 years; IV-9 age 29 years; IV-12 age 21 years; V-5 age 28 years; V-4 age 26 years, bottom panel). Families A and B with the *ATP11A* (NM_032189.3:c.332 2_3327+2dupGTC CAG GT) variant. **b** Full audiogram of Family B proband (PID IV-1; upper left). Audiograms (right ear series) of the proband, a sibling (PID IV-2) and cousin (PID IV-3) show variable configuration and progression of hearing loss (upper right and middle panels). **c** Full audiogram of the Family A proband (PID II-2; left). Audiogram of a sibling (PID II-3; right) shows symmetric high frequency hearing loss. Audiogram of parent PID I-2 reveals asymmetrical hearing loss; sloping moderate-to-profound in the left ear and no measurable hearing in the right ear. Since BC thresholds were symmetrical in all family proband audiograms, only one ear is displayed. *AC* air conduction, *BC* bone conduction

For the discovery phase, a six-generation family of Northern European descent was ascertained from the province of Newfoundland and Labrador (NL), Canada (Fig. 1). The proband (PID IV-7) presented at age 13 years with a progressive, sloping, bilateral SNHL and a family history consistent with autosomal dominant inheritance. Two Jewish Israeli families were studied at the Wolfson Medical Center, the Sheba Medical Center, and Tel Aviv University, serving as validation and consisted of a three-generation family (Family A) originating from Bukhara, Uzbekistan and a four-generation family with roots in Afghanistan (Family B) (Fig. 2). The proband in Family A (PID II-2) is a 46-year-old female with hearing loss. At the age of 39 years, she had normal low and mid-frequency hearing to 1 kHz and a sloping audiogram showing severe bilateral high-frequency SNHL. The proband in Family B (PID IV-1) is a 29-year-old female with hearing loss who was seeking genetic counseling to genetically diagnose the cause of familial hearing loss. At age 17 years she had normal low-mid frequency hearing and a high-frequency bilateral SNHL, sloping above 1 kHz. Hearing loss was evaluated in all families using standard pure tone measurements of air and bone conduction thresholds (Fig. 3a–c). Informed consent was obtained according to protocol #01.186 of the Human Research Ethics Board, St. John’s, NL, Canada and the Ethics Committee of Tel Aviv University and the Helsinki Committee of the Israel Ministry of Health.

### Targeted sequencing, genome-wide SNP genotyping and linkage analysis

For the discovery phase, genomic DNA was extracted from peripheral blood of the NL family using a modified salting out protocol (Miller et al. 1988) and screening was performed by bi-directional Sanger sequencing and analyzed using Mutation Surveyor Software (v5.0, SoftGenetics LLC State College, PA, USA). The NL proband was Sanger sequenced for all pathogenic variants causing hearing loss in this genetically isolated population, followed by select autosomal dominant genes matching the proband’s (PID IV-7) audiometric data with that of reference audioprofiles of 34 autosomal dominant deafness loci (Audiogene v4.0) (Hildebrand et al. 2009).

We next performed genome-wide single nucleotide polymorphism (SNP) genotyping using the Illumina 610Quad genotyping chip (Illumina Inc., San Diego, CA, USA) on multiple family members. Starting with a set of > 500,000 high quality SNP markers, informative SNPs (*n* = 17,407) were imported into Superlink (v1.7) (Fishelson and Geiger 2004) and linkage analysis performed under an autosomal dominant model with 99% penetrance and a disease allele frequency of 0.0025.

### Genome sequencing

Genomic DNA libraries were prepared on four affected (Fig. 1: PID IV-4, IV-6, IV-7, and IV-12) and two unaffected members of the NL family (PID IV-1 and IV-17) using the Lucigen Shotgun NxSeq AmpFREE Low DNA Library Kit (Cat. #14000-1, Lucigen Inc., Madison, WI, USA). Prepared libraries were loaded on an Illumina paired end 150 bp sequencing lane, and sequenced on the HiSeqX Sequencer (Illumina Inc., San Diego, CA, USA). Sequence reads were aligned to GRCh37 in both RefSeq and Ensembl reference genomes, and single nucleotide variants (SNVs) and insertions and deletions (INDELs) were called using GATK (v4.0). Structural chromosomal variants were called using Lumpy (v0.2.13) and SVtyper (v0.5.2), while a Bioconductor package, cn.MOPS (v1.26.0), was used for copy number variation (CNV) analysis. Variants were functionally annotated with SNPeff (v4.3T). We filtered for rare variants (MAF < 1%) residing within linked regions with a minimum of 20X coverage.

### Cascade sequencing, haplotype and in silico analysis

Candidate variants were amplified using a standard touchdown PCR protocol and sequenced in family members and compared to 202 SNHL probands and 326 ethnically matched controls from NL. Microsatellite markers and intergenic SNPs within linked regions were called with GeneMapper software (v4.0) and phased manually. Candidate splicing variants within exon–intron boundaries were analyzed in silico using MaxEnt, Human Splicing Finder (v3.1), and NNSPPLICE (v0.9) to predict their effect on RNA splicing.

### Experimental validation with patient-derived tissues

To validate variants predicted to alter RNA splicing, we extracted RNA from transformed B-cell lymphocytes from both affected and unaffected individuals (controls) from the NL pedigree (Fig. 1) using TRIzol-based methods (Thermo-fisher, Cat. #15596026). We prepared cDNA libraries with the Superscript VILO cDNA synthesis kit (Thermo-fisher, Cat. #11754050) followed by genomic DNA digestion (Turbo DNA-free kit, Invitrogen, Cat. #1907). Reverse transcription PCR (RT-PCR) was performed using a standard touchdown PCR protocol and primers that flanked splicing variants within two positional candidate genes (*ATP11A*: 5′ CCAGAGGGGTGTGAAGCA 3′ and 5′ CATCACACGAGCATTCCCAC 3′; *COL4A1*: 5′ GTTCACCTGGCTTACCTGGA 3′ and 5′ AAACCCACCTCACCCTTTG 3′). RT-PCR products were electrophoresed through 1.5% agarose Tris–Borate-EDTA gel and stained with SYBR Safe (Invitrogen, Cat. #S33102). For positional candidate genes expressing multiple transcripts, distinct bands were excised from the gel and cloned using the TOPO TA-Cloning Kit with One Shot TOP10 Chemically Competent *E. coli* (Invitrogen, Cat. #K457540) according to the manufacturer’s protocol. Clones were amplified using colony PCR (Costa and Weiner 2006) and Sanger sequenced. In addition, we tried to sequence full-length cDNA using long-range sequencing (Nanopore) and 5′ RACE with total RNA extracted from patients and controls.

### In vitro splice assay

To determine the effect of the *ATP11A* c.3322_3327+2dupGTCCAGGT variant, we performed a minigene splicing assay. A 554-bp genomic sequence, including exon 28 and flanking introns, was PCR amplified from wildtype and heterozygous DNA samples using specific primers introducing *Xho*I and *Bam*HI restriction sites. After digestion, PCR amplicons were cloned into the pET01 Exontrap vector (MoBiTec) using LigaFast DNA ligation kit (Promega). Wildtype and mutant minigene constructs were transfected into HEK293T cells, and RNA was extracted 48 h post-transfection using Trizol reagent. The synthesized cDNA was amplified using primers complementary to the 5′ and 3′ exons of the Exontrap vector. RT-PCR products were then visualized on gel, purified, and Sanger sequenced.

### Computer modeling of ATP11A

To determine the effect of the *ATP11A* c.3322_3327+2dupGTCCAGGT variant on protein structure, we used AlphaFold method (Jumper et al. 2021) through ColabFold local installation (Mirdita et al. 2021). AlphaFold produces a per-residue confidence score (pLDDT) between 0 and 100, which reliably predicts the Cα local-distance and estimates how well the predicted structure would agree with the experimental structure (Ruff and Pappu 2021). Some regions below 50 pLDDT may be unstructured in isolation.

Molecular graphics and analyses were performed with UCSF ChimeraX, developed by the Resource for Biocomputing, Visualization, and Informatics at the University of California, San Francisco, with support from National Institutes of Health R01-GM129325 and the Office of Cyber Infrastructure and Computational Biology, National Institute of Allergy and Infectious Diseases (Pettersen et al. 2021).

## Results

### Hearing loss phenotype

In the discovery family, sixteen members exhibit bilateral SNHL. Longitudinal audiograms on PIDs IV-4, IV-6, IV-7, IV-9, IV-12, IV-14, V-4, V-5, V-6, V-9, and V-11 reveal a progressive loss with variable onset and configuration, with auditory profiles ranging from high-frequency sloping loss to low-mid frequency and flat configurations (Fig. 3a). For example, the proband (PID IV-7) has a sloping high-frequency hearing loss whereas his son (PID V-6) has a relatively flat configuration. Longitudinal audiograms for a cousin (PID V-4) reveal a low-mid frequency rising configuration. The proband (PIDs IV-7) and his father (PID III- 5) were identified with hearing loss in their first decade. In contrast, two members (PIDs V-6, VI-1) failed newborn screening. Other members reported hearing loss in the 2nd or 3rd decade (age 28 for PID V-5, Fig. 3a). The course of hearing deterioration is also variable. Hearing sensitivity declined into the severe to profound range by the 2nd decade for some members (PIDs V-4, V-6) and in the 3rd–6th decade for others (PIDs IV-12, IV-7, IV-9, IV-14, IV-6).

The SNHL in Family A (HL1515) is post-lingual onset, progressive, with variable severity, starting mostly in high frequencies and deteriorating with age (Fig. 3c). We also observed earlier onset from one generation to the other, as the grandmother, (PID I-2), and her two daughters, (PIDs II-2 and II-3), reported onset in their late teens, but the onset of SNHL in the grandchildren, (PIDs III-2 and III-3) was in early childhood. SNHL in Family B is also progressive with variable onset and configuration. Twelve members have bilateral SNHL (PIDs II-2, III-4, III-6, III-8, III-10, IV-1, IV-2, IV-3, IV-4, IV-5, IV- 12, and IV-13). Pure tone audiogram data for the proband (PID IV-1) (Fig. 3b), the proband’s sibling (PID IV-2) and cousin (PID IV-3) show variability in the audiometric configuration and degree of hearing loss. Longitudinal audiograms for the proband PID IV-1 (Fig. 3b) show sloping high-frequency hearing loss and progression over three years. Consecutive audiograms for PID IV-2 show all frequency loss and progression over six years (Fig. 3b). A single audiogram for PID IV-3 at age 40 shows moderate low and mid-frequency loss, and a peak at 2 kHz with severe high-frequency loss (Fig. 3b). Hearing loss was present in the proband (PID IV-1) by the early 2nd decade; onset is reported during the first decade for a sibling (PID IV-2) and two other members (PID IV-3 and IV-12).

### Linkage analysis links hearing loss to *DFNA33* (13q34)

The NL proband (PID IV-7) screened negative for all genetic variants we previously identified in this genetic isolate (Table S1). Furthermore, although audiometric data analysis yielded positive gene matches, bi-directional Sanger sequencing of targeted genes based on audioprofiles yielded wild-type sequence (data not shown). Genome-wide SNP genotyping and two-point linkage analysis yielded statistically significant linkage (LOD = 4.77; Table 1) to a 3.6 Mb region on chromosome 13q34 overlapping *DFNA33* (13q34-13qter) (Bonsch et al. 2009).

**Table 1:** Genomic regions with maximum observed LOD > 1.5 in a two-point linkage analysis under a dominant model with 99% penetrance and a disease allele frequency of 0.0025

Chr	LOD	Start			End			Region size (Mb)
		SNP	cM	Genomic position	SNP	cM	Genomic position	
1	2.54	rs591979	85.54	61,368,955	rs9629017	87.62	62,083,960	0.72
1	2.14	rs6593523	101.44	76,486,908	rs1360878	101.82	76,749,088	0.26
1	1.87	rs1325278	109.17	85,400,182	rs817485	109.42	85,573,095	0.17
5	1.56	rs253604	161.03	155,960,089	rs6892282	163.77	159,360,485	3.40
5	1.55	rs11954477	167.38	163,374,345	rs253537	169.34	164,600,485	1.23
**13**	**4.77**	**rs872484**	**117.51**	**110,708,368**	**rs9324254**	**128.64**	**114,312,000**	**3.60**
19	1.83	rs4527136	25.72	8,186,519	rs2042300	26.79	8,580,602	0.39
19	1.83	rs2060260	38.32	15,704,783	rs1558139	38.55	15,997,564	0.29

Genomic positions are captured using GRCh37

Bold represents the highest LOD score and evidence of statistically significant linkage of hearing loss to chromosome 13

*LOD* logarithm of the odds (to the base 10), *Chr* chromosome, *cM* centimorgan, *SNP* single nucleotide polymorphism, *Mb* megabase

### Haplotype analysis reduces the number of candidate variants

**Fig. 4:**
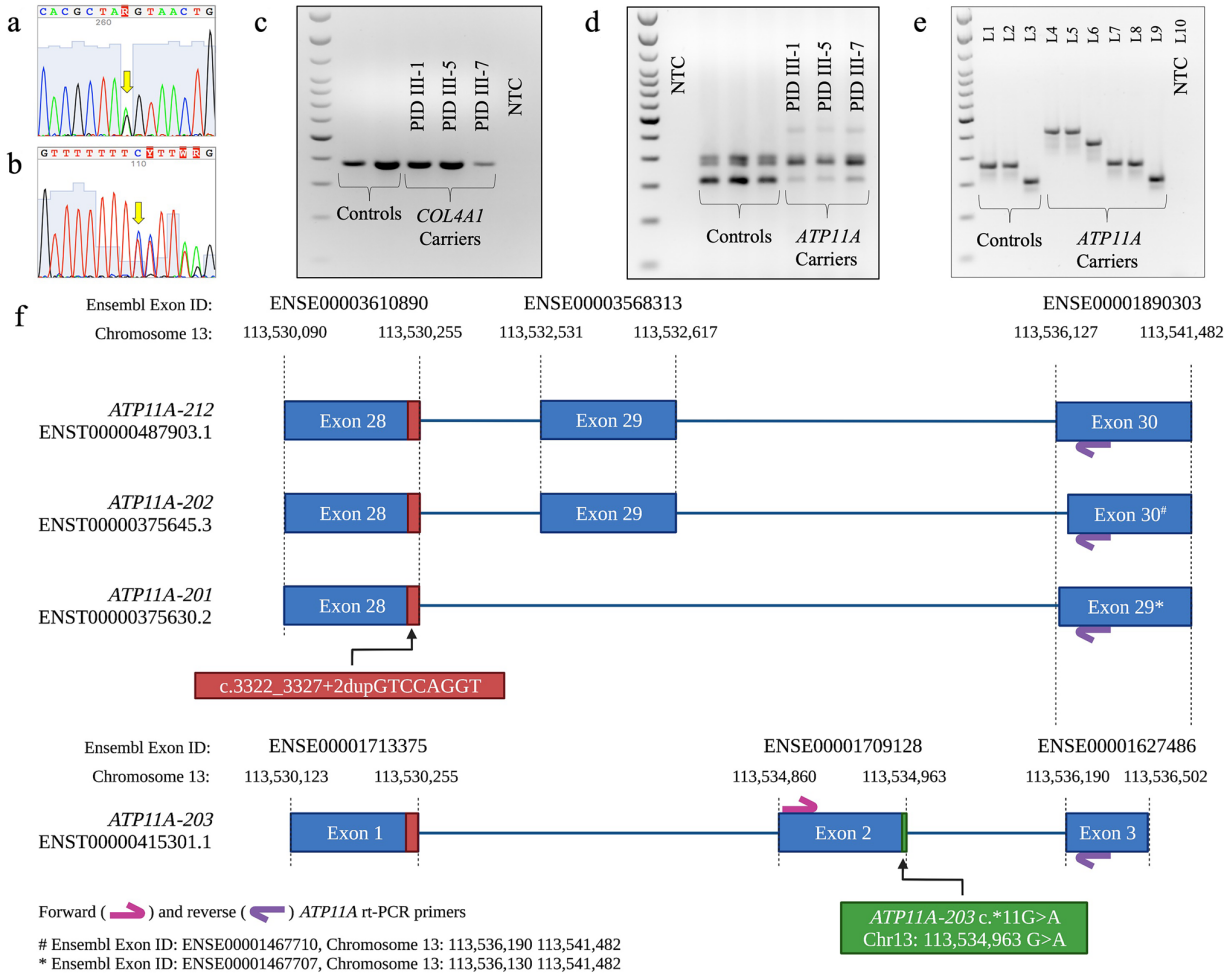
Experimental design and RNA data analysis of candidate splicing variants derived from B-cells in unaffected (controls) and patients (*COL4A1* GRCh37 NM_001845.6:c.3326-7dupT and *ATP11A* chr13:113534963G>A carriers). **a** Electropherogram illustrating a heterozygous *ATP11A* (NC_000013.11:chr13:113534963G>A) variant (Fig. 1: PID: III-1). **b** Electropherogram illustrating a heterozygous *COL4A1* (NC_000013.11:g.110174539dupT; NG_011544.2:g.137617dup; NM_001845.6:c.3326-7dupT) variant (Fig. 1: PID: III-1). **c** RT-PCR analysis of the *COL4A1* c.3326-7dupT variant revealed a single ~ 280 bp amplicon in unaffecteds (controls) and *COL4A1* c.3326-7dupT carriers (PID III-1, III-5, and III-7). **d** RT-PCR analysis of the 3′ region of *ATP11A* flanking the GRCh37 chr13: 113534963G>A variant revealed multiple bands in both unaffected (controls) and *ATP11A* GRCh37 chr13: 113534963G>A carriers, which were TA-Cloned (**e**). The TA-clones of lower-molecular weight from both unaffected controls (lanes 1–3) and *ATP11A* carriers (lanes 7–9) mapped to wild-type *ATP11A* sequence. The three higher-molecular weight bands (lanes 4–6) that were only observed in *ATP11A* carriers revealed retained intronic sequence. NTC: non-template control. The 100 bp ladder is indicated on the left of the gels with the densely stained bands representing 600 bp and 1500 bp. **f** Schematic of *ATP11A-203* aligned against the 3′ region of the three longest *ATP11A* isoforms (*ATP11A-201*, *-202*, *-212*). The NL *ATP11A* variant chr13:113534963G>A (green) is positioned at the terminating bp of *ATP11A-203* exon 2 (ENSE00001709128), while the Jewish Israeli variant (red) is located at the end exon 28 of *ATP11A-201*, *-202*, *-212* (ENSE00003610890) and exon 1 (ENSE00001713375) of *ATP11A-203*. The location of the forward (pink arrow) and reverse (purple arrow) primers are also indicated with their respective exon locations. *L* lane

Genome sequencing of selected NL members yielded an average coverage of 44X with 94% of the genome covered at 25X (Table S2). No structural chromosomal rearrangements or CNVs were detected (data not shown). Of the 15,071 variants identified in affected family members, 51 resided within the 3.6 Mb disease interval on 13q34 (Table S3). Two candidate splicing variants, one novel: *ATP11A* (NC_000013.11:chr13:113534963G>A; ENST00000415301.1:ATP11A-203:c.*11G>A; Fig. 4a) and one rare: *COL4A1* (NC_000013.11:g.110174539dupT; NG_011544.2:g.137617dup; NM_001845.6:c.3326-7dupT; Fig. 4b) were both absent in NL population controls. In silico tools predict that *COL4A1* c.3326-7dupT does not disrupt splicing, which we confirmed by RNA analysis (Fig. 4c). Furthermore, during the transcriptional analysis, we recruited two unaffected family members (PID III-4 and V-2) that harbored key cross over events and reduced the disease interval to 769 kb, excluding the *COL4A1* c.3326-7dupT (Fig. 1). Subsequently, 202 NL probands with hearing loss were screened wild-type for *ATP11A* chr13:113534963G>A.

### RNA analysis of the NL family reveals several *ATP11A* products and an extra 3′ coding exon

The *ATP11A* gene encodes a member of the family of P4-ATPase proteins and has 17 transcripts (Human GRCh37 Ensembl 93 build (Yates et al. 2016)), most of which are incompletely annotated. The *ATP11A* gene has three long isoforms (Fig. 4f). Ensembl reports *ATP11A-201* (ENST00000375630.6), *ATP11A-202* (ENST00000375645.7) and *ATP11A-212* (ENST00000487903.5), which also includes the two RefSeq transcripts: *ATP11A-202* (NM_015205: isoform a: 8795 bp) and *ATP11A-201* (NM_032189: isoform b: 8768 bp). In silico algorithms predict this novel *ATP11A* variant found in the NL family has the potential to disrupt a canonical donor splice site (Medium Impact; Table S4). According to NNSPLICE analysis, the *ATP11A* variant is predicted to activate a cryptic donor splice site 153 bp downstream of the canonical donor splice site (Table S5). RT-PCR analysis of the 3′ region of *ATP11A* (flanking the GRCh37 chr13:113534963G>A variant) in unaffecteds and *ATP11A* GRCh37 chr13:113534963G>A carriers (PID III-1, III-5 and III-7) revealed multiple products (Fig. 4d). RT-PCR amplification revealed three amplicons shared by unaffected and affected family members, as well as three additional amplicons of higher-molecular weight amplicons in *ATP11A* carriers (Fig. 4e).

**Fig. 5:**
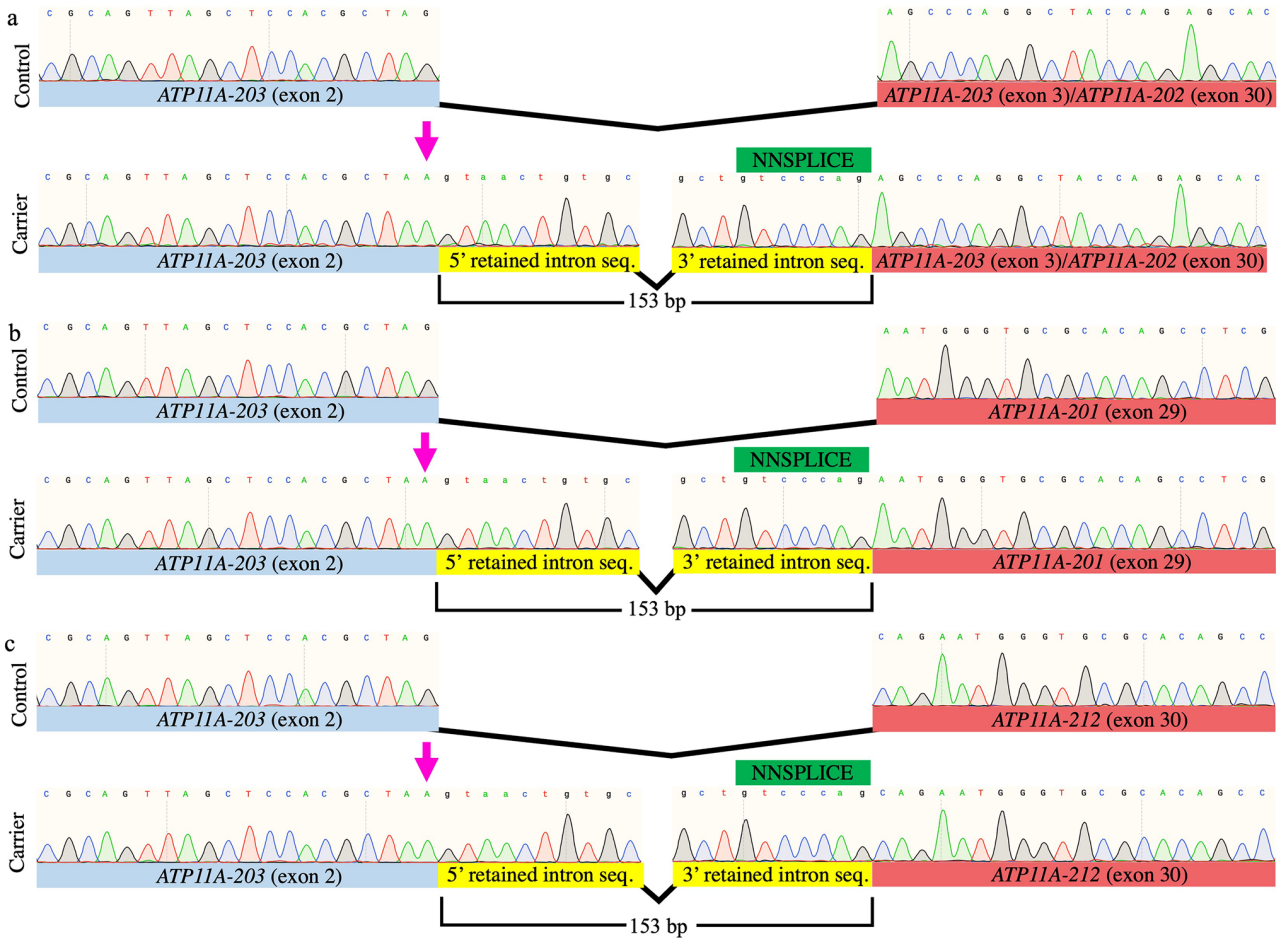
Sequencing of TA-cloned *ATP11A* RT-PCR products from healthy controls and affected family members. The genomic position of the *ATP11A* variant (NC_000013.11:chr13:13534963G>A) is indicated by a pink arrow and activates a cryptic splice site causing intron retention of 153 bp. For visualization purposes, 20 bp of the shared 104 bp exon and the first 20 bp of the next known 5′ exon are displayed. Cryptic spliced region is represented by the first and last 10 bp of the 5′ and 3′ ends of the retained intronic sequence, respectively. **a** Upper electropherogram: sequence present in both controls and affected family members (Fig. 4e, lanes 3 and 9). The first 20 bp of this sequence aligns to *ATP11A-203* (exon 2) and the following 20 bp aligns to both *ATP11A-203* (exon 3) and *ATP11A-202* (exon 30). Lower electropherogram: sequence harboring the *ATP11A* variant found in affected family members that activates a cryptic donor splice site 153 bp downstream (Fig. 4e, Lane 6). **b** Upper electropherogram: sequence found in both control and affected family members (Fig. 4e, lanes 2 and 8) that aligns to *ATP11A-203* (exon 2) and *ATP11A-201* (exon 29). Lower electropherogram: sequence harboring the *ATP11A* variant found in affected family members that activates a cryptic donor splice site 153 bp downstream (Fig. 4e, lane 5). **c** Upper electropherogram: sequence present in both controls and affected family members (Fig. 4e, lanes 1 and 7) that aligns to *ATP11A-203* (exon 2) and *ATP11A-212* (exon 30). Lower electropherogram: sequence harboring the *ATP11A* variant found in affected family members that activates a cryptic donor splice site 153 bp downstream (Fig. 4e, Lane 4). Control: wild-type *ATP11A* cDNA sequence, found in both control and affected family members. Carrier: *ATP11A* variant (chr13:113534963G>A) cDNA sequence, which is exclusive to affected family members. Green box: DNA motif predicted by NNSPLICE as the most probable cryptic donor splice site, in the absence of the natural splice site (Table S5). Uppercase font denotes exonic sequence, and lowercase font indicates retained intronic cDNA. Pink arrow denotes the *ATP11A* chr13:113534963G>A variant

Sequencing of cloned RT-PCR products in unaffected and *ATP11A* carriers show they all contained a 104 bp exon (i.e., NC_000013.11), which map to exon 2 of the *ATP11A-203* transcript (ENST00000415301.1; Fig. 4) and is consistent with the common lower-molecular weight bands (Fig. 4e). Downstream of the shared 104 bp exon, three unique sequences were observed, aligning to exon 3 of *ATP11A-203* and exon 30 of *ATP11A-202* (ENST00000375645.7, lower-molecular weight), exon 29 of *ATP11A-201* (ENST00000375630.6; NM_032189) and exon 30 of *ATP11A*-*212* (ENST00000487903.5; NM_015205; higher-molecular weight; Figs. 4e, 5). Whether or not the 104 bp exon represents the penultimate exon in *ATP11A*-201 (ENST00000375630.6; NM_032189), *ATP11A*-*202* (ENST00000375645.7) and *ATP11A*-*212* (ENST00000487903.5; NM_015205) or represents a novel transcript is unknown (Fig. 5). Interestingly, this 104 bp sequence is similar (61.5% conserved) to exon 29 (*ATP11A-201*) in the mouse (Fig. S1), which is consistent with location of the 104 bp spliced region from our RNA analysis (Fig. 5). Full-length sequencing of these distinct RNA transcripts was attempted several times but failed to confirm which of the many *ATP11A* isoforms are being expressed in blood.

### RNA validation of aberrant splicing in patient-derived tissues of the NL family

NNSPLICE analysis predicted that the *ATP11A* GRCh37 chr13:113534963G>A variant may activate a cryptic splice site 153 bp downstream of the canonical donor splice site. Therefore, we designed exonic primers that span the intron/exon boundaries of exon 2 and 3 of *ATP11A-203* (Fig. 4f). Sequencing of cloned RT-PCR products in *ATP11A* GRCh37 chr13:113534963G>A carriers show they retain 153 bp of intronic sequence at the 3′UTR of *ATP11A* and is consistent with the three aberrant higher-molecular weight products (Figs. 4e–f and 5). This intronic sequence was not observed in unaffected family members tested (Figs. 4e and 5). Whether or not the 153 bp extends exon 29 in the putative *ATP11A-201* isoform (Fig. 5a) and exon 30 in the putative *ATP11A-202/212* isoforms (Fig. 5b), or represents a novel transcript is unknown (Fig. 5). As the RT-PCR analysis and sequencing was restricted to the 3′ region of *ATP11A* (flanking the GRCh37 chr13:113534963G>A variant), full-length sequencing is required to confirm the variant effect on *ATP11A* isoforms. Unfortunately, long-range sequencing (Nanopore) and 5′ RACE failed to capture full-length *ATP11A* cDNA in peripheral blood. The *ATP11A* variant is classified as likely pathogenic by ACMG criteria (PM2, PP1_S, PP3) (Richards et al. 2015; Oza et al. 2018).

### Unrelated families from Israel with a novel duplication in exon 28 of *ATP11A*

Whole exome sequencing performed at Macrogen Europe revealed a duplication of 8 nucleotides, c.3322_3327+2dupGTCCAGGT in the *ATP11A* gene (NM_032189.3) in the Jewish Israeli family, Family A. The duplication co-segregates fully with dominant hearing loss in five affected and three unaffected family members. Genomic DNA from the proband (PID IV-1) and two cousins (PIDs IV-3, IV-12; Fig. 2) in Family B underwent clinical exome sequencing (CeGaT Laboratory) and screened negative for variants in DFNA genes but were all heterozygous for the same duplication that was identified in Family A (sequence not shown). This variant has not been seen in 5000 previous exome analyses at the CeGaT Laboratory or previously described in the gnomAD database.

### Minigene splicing assay of the variant in the Jewish Israeli families validates normal splicing but uncovers an out-of-frame insertion

**Fig. 6:**
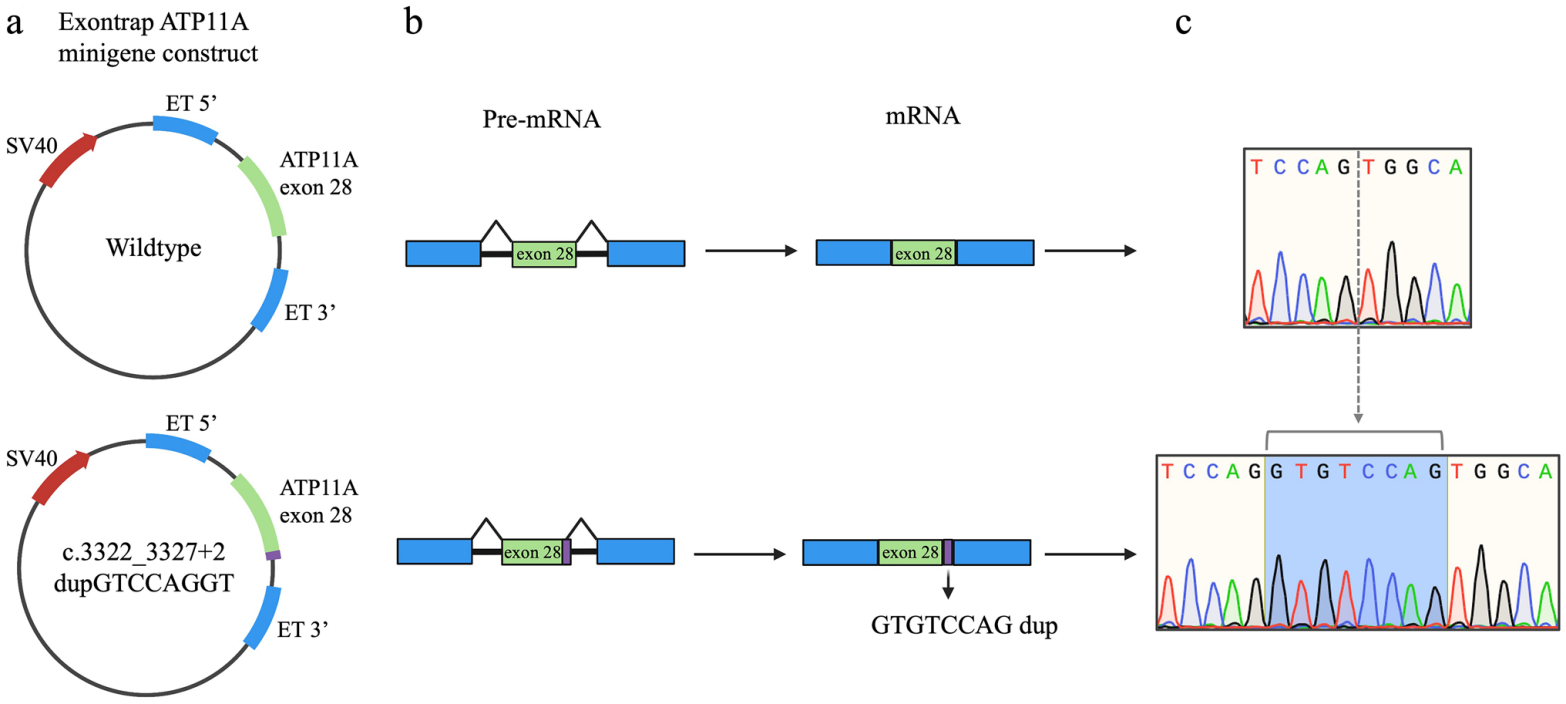
Minigene assay of the c.3322_3327+2dupGTCCAGGT variant. **a** Exontrap-ATP11A minigene constructs showing cloned *ATP11A* (green) wildtype (upper panel) and duplication-containing (lower panel, green with duplication in purple) exon 28 regions with flanking intronic sequence that is inserted between two artificial exon trapping (ET) exons (blue). **b** Schematic showing pre-mRNA splicing to produce mature mRNA of the wildtype (upper panel) and duplication (lower panel) that indicates normal splicing but confirms insertion of 8 bp due to the duplication that was validated with Sanger sequencing (**c**)

The splice site duplication located at the 28 exon–intron boundary includes duplication of the last 6 bp of exon 28 and the 2 first bp of intron 28, which suggests that the exon/intron boundary is not affected but removed 8 bp downstream, and the splice pattern is expected to be conserved (Fig. 6). Thus, the insertion is predicted to extend exon 28 by 8 bp, including the 2 intronic nucleotides (gt), followed by the 6 last exonic nucleotides (GTCCAG), leading to a frame shift at amino acid 1110 (82 amino acids before the end of the protein) and a stop codon 43 amino acids downstream, p.Asn1110Valfs43Ter. This prediction was validated by a minigene assay that did not show aberrant splicing; however, sequencing of the normal and mutant minigene products demonstrated that the insertion adds 8 bp to exon 28, as described above.

### Computer modeling of the *ATP11A* c.3322_3327+2dupGTCCAGGT

Modeling of the wildtype (WT) and mutant molecules showed that the majority of residues in both molecules are well organized, with very accurate per-residue prediction (pLDDT values > 80 for 95% of the residues). There are also three more flexible, less structured loops with pLDDT values < 50 (residues and 1–20, 442–459, 487–500 and 739–752), and long fully flexible C-terminus region starting from Q1114 in WT and T1120 in mutant which have pLDDT values < 30 and thus predicted to be complete disordered. This result suggests that the C-terminus region is intrinsically disordered and possibly adopts a more structured conformation upon interaction with a partner protein or cell membrane, or cellular localization, etc. (Fig. S2). Additionally, we found that the WT and mutant isoforms have a very different pattern of charged distribution along the C-terminus region (Fig. S2). While both C-terminal regions have a net charge of + 6, the WT includes 11 positively charged residues (4 Arg, 2 Lys and 5 His) and 5 negative residues (3 Glu and 2 Asp), the mutant isoform includes 6 positively charged residues (5 Arg and 1 His) and strikingly no negative residues. This is indeed very intriguing and suggests an alteration of the electrostatic pattern in the Cterm upon variant, with the WT having a much longer (81 aa), highly charged, His rich C-terminal tail very different from the shorter (42 aa), Arg rich C-term tail of MUT isoform nude of negative residues.

As the C-terminus region of ATP11A has been shown to be critical for Ca^2+^-dependent endocytosis or polarized localization at the plasma membrane (Okamoto et al. 2020), we believe our modeling of the WT and mutant molecules with AlphaFold suggest then important differences in the C-terminus charge organization may hint about the impaired localization of the mutant isoform. The *ATP11A* c.3322_3327+2dupGTCCAGGT variant is classified as pathogenic by ACMG criteria (PS1, PS4_P, PM2, PM4, PP1_S, PP3) (Richards et al. 2015; Oza et al. 2018).

## Discussion

We report the first family, a Canadian family of Northern European descent (NL family) with autosomal dominant SNHL to be linked to *DFNA33* since the locus was mapped in 2009 (Bonsch et al. 2009). Fine mapping and sequencing revealed a novel splice variant in the 3′ region of *ATP11A* gene. We also recruited two Jewish Israeli families (unrelated) and identify a second, novel variant in the 3′ region of *ATP11A*. Splice variants in the 3′ region of *ATP11A* cause a bilateral, progressive SNHL with variable onset and configuration. Of note, the intrafamilial variability among individuals in Family A with respect to onset could be explained by (1) large variability in onset of this variant, or (2) the hearing loss not being noticed in younger ages in the older generations (before the era of routine hearing screening tests) as hearing loss starts in high frequencies, not so much affecting speech and normal life, or (3) anticipation revealed in earlier onset from generation to generation. The asymmetry in the older family member, PID I-2, could also have several explanations including (1) an additional layer of variability that includes asymmetry between the ears or (2) right dead ear as a result of another reason, e.g., viral cause, phonal trauma, etc., and only the left ear is displaying the ATP11A phenotype or (3) the deterioration with age is not symmetric, starting with one ear and deteriorating faster than the other, but with time the other ear might reach the same degree of severity. This phenomenon is common and seen with other progressive deafness genes as well. The hearing loss in the German *DFNA33* family is quite similar to *ATP11A* variant carriers in both the NL and the Israel families. Although we cannot be certain that the *ATP11A* gene is *DFNA33*, it was noted to be a functional candidate based on mouse studies (Bonsch et al. 2009). Although a decade has passed since *DFNA33* was mapped to chromosome 13q34-qter, no other families have been reportedly mapped to this locus. Although other phospholipid flippases (P4-ATPases) are associated with syndromic forms of hearing loss, this study documents the first association of *ATP11A* with a highly penetrant Mendelian phenotype.

Pathogenic variants in specific phospholipid flippases have been linked to familial intrahepatic cholestasis (Bull et al. 1998), severe neurological and motor disorders (Martin-Hernandez et al. 2016) and congenital hemolytic anemia (Arashiki et al. 2016). P4-ATPases comprise a subfamily of P-type ATPases that flip phospholipids from the exocytoplasmic to the cytoplasmic leaflet of cell membranes that both generates and maintains phospholipid asymmetry and are organized into five subclasses based on the sequence similarity of their catalytic subunits. These proteins contain a large catalytic or α-subunit composed of a nucleotide binding domain (N-domain), a phosphorylation domain (P-domain), and an actuator domain (A-domain) as well as a membrane domain (M-domain) comprised of 10 transmembrane segments (Andersen et al. 2016) and forms a heteromeric complex with CDC50 (Bryde et al. 2010). Furthermore, mammalian P4-ATPases can contain an extended C-terminal segment implicated in protein folding and regulation of its activity (Chalat et al. 2017).

The ATP11A protein specifically transports phosphatidylserine (PS) and phosphatidylethanolamine (PE) across cell membranes, is ubiquitously expressed in various tissues and deletion of in *atp11a* in mice results in lethality during embryogenesis (Segawa et al. 2016; Wang et al. 2018). Of the 17 annotated *ATP11A* transcripts, the putative *ATP11A* GRCh37 chr13:113534963G>A variant mapped to a short Ensembl isoform containing only three exons. RT-PCR and cloning analysis of the 3′ region of *ATP11A* in unaffecteds and *ATP11A* carriers revealed multiple products, three products in unaffecteds and these three products plus three extra higher-molecular weight products in family members with hearing loss. The *ATP11A* splicing variant is predicted to activate a cryptic donor splice site 153 bp downstream, which extends the 3′UTR, was confirmed with RNA analysis. This study also uncovered the *ATP11A* c.3322_3327+2dupGTCCAGGT variant in two unrelated Jewish Israeli families, one originating from Bukhara, Uzbekistan, and the other from Afghanistan. As Afghani Jews originate either from Persia or from Bukhara, we suggest the same founder of the variant in both families, although haplotype analysis was not performed.

The disease mechanism, how variants in the *ATP11A* gene cause hearing loss, is unclear. It is known that the 3′ end of the mammalian P4-ATPases are important for protein folding and regulation of its activity (Chalat et al. 2017). It is likely that pathogenic splicing variants act via dominant-negative or haploinsufficiency mechanism.

Through combining single-cell and long-read RNA-seq technologies, Ranum et al (2019) has recently revealed clusters of genes that define inner hair cells, outer hair cells and Deiter cells, and identified many heretofore unrecognized exons, alternative splicing diversity and isoform abundance in hearing loss genes. According to the Molecular Otolaryngology and Renal Research Laboratories database, *ATP11A-203* (exon 2) murine homolog, *Atp11a* (exon 29) was expressed in inner hair cells, outer hair cells and Deiter cells. Most importantly, *Atp11a* was identified as a Deiter cell defining gene that exhibits moderate expression during mouse embryonic development, followed by a marked increase in expression after birth (Shen et al. 2015). Animal models for *Atp11a*-induced hearing loss could help elucidate the molecular mechanisms, as recent work has shown that *atp11a* is also expressed in the zebrafish ear (Hawkey-Noble et al. 2020). Taken together this evidence suggests that the genomic complexity of *ATP11A* expression is similar in mouse and humans.

This study highlights the power of robust linkage analysis combined with genome sequencing to identify private and unannotated variants as disease-associated alleles. Over the ten-years of study on the NL family, we have benefited from ongoing recruitment and comprehensive phenotyping which eventually identified key recombinations to the disease-associated haplotype. The use of haplotype analysis reduced the number of candidate variants, which made the problem tractable. Given the complexity in genomes, a comprehensive bioinformatics pipeline targeting all known transcripts is essential as is the need to experimentally validate in silico predictions in patient-derived tissues.

Limitations of this study include a lack of insight as to protein function, specifically with respect to hearing loss. In addition, given that the complete *ATP11A 201/202/212* transcripts were not successfully sequenced from peripheral blood, it is uncertain if the penultimate exon is present in these *ATP11A* transcripts or represents the 3’ end of a novel transcript. Likewise, it is uncertain if the splicing variant in *ATP11A* carriers causes the retention of 153 bp in the penultimate exon of *ATP11A 201/202/212* transcripts or represents the 3′ end of a novel transcript.

Future directions for this study include in vitro and in vivo functional characterization of the molecular mechanisms of the two *ATP11A* variants. Given that ATP11A specifically transports PS and PE across cell membranes, the deregulation of transport could redistribute PS to the extracellular side of plasma membrane flagging cells for their recognition, phagocytosis, and ultimate degradation by phagocytes (Schroit et al. 1985). Phagocytic signals such as PS at the cell surface are known pharmaceutical targets (Birge et al. 2016), so there is potential that hearing loss due to *ATP11A* could be pharmaceutically treated. It will be important, if possible, for the original German family used to map *DFNA33* be sequenced to be certain that *ATP11A* is *DFNA33*.

## Web resources

NNSPLICE: https://www.fruitfly.org/seq_tools/splice.html.

Hereditary Hearing Loss Homepage: http://hereditaryhearingloss.org.

Audiogene (v4.0): https://audiogene.eng.uiowa.edu/.

CeGaT Lab: https://www.cegat.de/en/diagnostics/exome-diagnostics/the-best-possible-exome/.

Genome Aggregation Database (gnomAD): https://gnomad.broadinstitute.org/.

Ensembl: https://useast.ensembl.org/index.html.

International mouse phenotyping consortium: http://www.mousephenotype.org/data/genes/MGI:135473524.

miRBase: the microRNA database: http://www.mirbase.org/.

Molecular Otolaryngology and Renal Research Laboratories database: https://morlscrnaseq.org/.

## Supplementary Information

Below is the link to the electronic supplementary material.

Supplementary file1 (PDF 370 kb)

## Data Availability

The datasets generated during and/or analyzed during the current study are available from the corresponding author on reasonable request.
