# Supplementary material for: Autosomal dominant non-syndromic hearing loss maps to DFNA33 (13q34) and co-segregates with splice and frameshift variants in ATP11A, a phospholipid flippase gene
Source: Hum Genet. 2022 Mar 12;141(3-4):431–44. doi: 10.1007/s00439-022-02444-x (PMC9035003; doi:10.1007/s00439-022-02444-x)
Supplement: Supplementary file 1 — Supplementary file1 (PDF 370 kb) [file 439_2022_2444_MOESM1_ESM.pdf]

TITLE: Autosomal dominant non-syndromic hearing loss maps to *DFNA33* (13q34) and co-segregates with splice and frameshift variants in *ATP11A,* a phospholipid flippase gene.

Justin A. Pater,[1,2] Cindy Penney,[1] Darren D. O'Rielly,[1,3] Anne Griffin,[1] Lara Kamal,[4] Zippora Brownstein,[4] Barbara Vona,[5,6] Chana Vinkler,[4] Mordechai Shohat,[7] Ortal Barel,[7] Curtis R. French,[1] Sushma Singh,[8] Salem Werdyani,[1] Taylor Burt,[1] Nelly Abdelfatah,[1] Jim Houston,[1] Lance P. Doucette,[1] Jessica Squires,[1] Fabian Glaser,[9] Nicole M. Roslin,[10] Daniel Vincent,[11] Pascale Marquis,[12] Geoffrey Woodland,[1] Touati Benoukraf,[1] Alexia Hawkey-Noble,[1] Karen B. Avraham,[4] Susan G. Stanton,[8*] Terry-Lynn Young[1, 3*]

[1]Faculty of Medicine, Memorial University, 300 Prince Phillip Drive, St. John's, NL, Canada.

[2]Dana-Farber Cancer Institute, Harvard Medical School, Boston, MA, USA.

[3]Centre for Translational Genomics, Memorial University, 300 Prince Phillip Dr., St. John's, NL, Canada.

[4]Department of Human Molecular Genetics and Biochemistry, Faculty of Medicine and Sagol School of Neuroscience, Tel Aviv University, Tel Aviv 6997801, Israel.

[5]Institute of Human Genetics, University Medical Center Göttingen, Göttingen, Germany.

[6]Institute for Auditory Neuroscience and InnerEarLab, University Medical Center Göttingen, Göttingen, Germany.

[7]Bioinformatic Center, Cancer Research Institute, The Wohl Institute for Translational Medicine, Sheba Medical Center, Tel-Hashomer, Israel, and Sackler School of Medicine, Tel Aviv University, Israel.

[8]Communication Sciences and Disorders, Western University, Elborn College, 1201 Western Road, London, Ontario, Canada

[9]The Lorry I. Lokey Center for Life Sciences and Engineering, Technion - Israel Institute of Technology, Haifa, Israel[10]The Centre for Applied Genomics, The Hospital for Sick Children, Peter Gilgan Centre for Research and Learning, 686 Bay Street, Toronto, ON, Canada

[11]Genome Quebec Innovation Centre, McGill University, 740 Dr. Penfield Avenue, Montréal, QC, Canada

[12]Canadian Centre for Computational Genomics, McGill University and Genome Quebec Innovation Center, 740 Dr. Penfield Avenue, Montréal, QC, Canada

*Co-Senior author

Corresponding Author: Terry-Lynn Young, Tel: (709) 864-6512, Mobile: (709) 699-3502, email: tlyoung@mun.ca

Conservation: 61.5%

```
           1       10        20        30        40        50        60        70        80        90        100 104
           |--------+---------+---------+---------+---------+---------+---------+---------+---------+---------+---|
   Human:  AGGGGTGTGAAGCACAAGGCTCCAGTCCAGGCCGCACAGAGCAGCGATGGGCCCCTCCTGAAGGACCTCCTACGGCGGCCAAGGCGCAGTTAGCTCCACGCTAG
   Mouse:  CAGAGCACCCGACTCAAAGCATTGGCCGATGCCACCTCGAACAGCGCCAGGCCCCTGCTGAAGGACTTCTTACCGCAGCCAAGACATGTCTAGCCCAACGCTAG
Consensus  aaGaGcaccaaaCaCAAaGCaccaGcCcAgGCCaCacaGAaCAGCGacaGGCCCCTcCTGAAGGACcTCcTACcGCaGCCAAGaCacagcTAGCcCaACGCTAG
```

Figure S1. Multiple species alignment of human (chr13: 113,534,860-113,534,963, *ATP11A-203*, ENST00000415301, Exon 2: 104bp) and mouse (chr8: 12,863,758-12,863,862, *ATP11A-201*, ENSMUST00000033818, Exon 29: 104bp) using MultAlin (http://multalin.toulouse.inra.fr/multalin/)

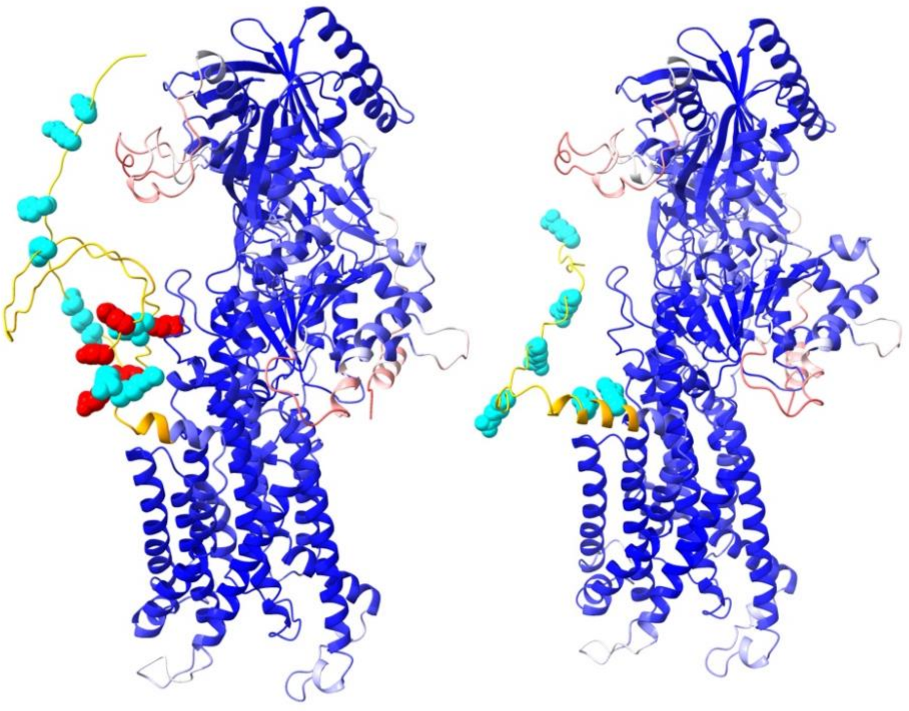

Figure S2. AlphaFold 3D structure models for the WT (left panel) and mutant isoforms of ATP11A (right panel). Both structures are colored by AlphaFold confidence measure pLDDT from blue (high accuracy) to red (low accuracy). The C-term region (GLN 1109 to the end) in both structures is colored gold, having a very low pLDDT. C-term Arg, Lys and His are shown in CPK and colored cyan, while Glu and Asp residues are shown in CPK and colored red.

Table S1. Recurrent Hearing Loss Mutations in Newfoundland

Gene	Accession No.	Variant	DFN Locus	Locus (Cytogenetic)
CLDN14	NM_144492	c.488C>T	*DFNB29*	21q22.13
TMPRSS3	NM_024022	c. 207delC	*DFNB8/10*	21q22
		c. 268 G>C		
		c. 782+3delGAG		
		c. 757 A>G		
		c. 612-2insTA		
WFS1	NM_006005.3	c. 2146 G>A	*DFNA6/38*	4p16.3
		c. 1832 A>G		
PCDH15	NM_033056	c. 1583 T>A	*DFNB23*	10p11.2-q21
		c. 1590 + 20 A>G		
KCNQ4	NM_004700	c. 806delCCT	*DFNA2A*	1p34
GJB2	NM_004004	c.-23+1G>A	*DFNA3A/B1*	13q11-q12
		c. 35delG		
		c. 101 T>C		
		c. 229 T>C		
		c. 249 C>G		
		c. 167delT		
GJB6	NM_006783	delD1351830	*DFNA3B*	13q12
		delD1351854		
GJB3	NM_024009.2	c. 109 G>A	*DFNA2B*	1p35.1
SMPX	NM_014332.2	c. 99delC	*DFNX4*	Xp22
COCH	NM_004086.2	c. 151 C>T	*DFNA9*	14q12-q13
TECTA	NM_005422	c. 26557 A>G	*DFNB21*	11q
TMC1	NM_138691	c. 421C>T	*DFNA36*	9q13-a21

Table S2. Coverage Statistics

Sample	PID IV-1	PID IV-4	PID IV-6	PID IV-7	PID IV-12	PID IV-17	Average
**Total Reads**	486,564,501	484,712,766	487,129,511	460,365,839	487,377,920	476,685,800	480,472,723
**10X**	96.34%	96.36%	96.37%	96.81%	96.87%	96.32%	96.51%
**25X**	94.81%	94.78%	94.83%	91.30%	92.30%	94.73%	93.79%
**50X**	27.04%	26.33%	27.44%	20.48%	30.61%	24.79%	26.10%
**Mean Coverage**	44.48	44.31	44.57	42.07	44.78	43.92	44.02

Table S3. Rare variants (<1% MAF) residing within the linked 3.6 Mb region.

Chr	Start	End	Gene	Variant	dbSNP	Impact	MAF (%)
13	98300574	98300575	*PSMA6P4-RP11-120E13.1*	n.98300575G>C	None	Low	-
13	98300580	98300581	*PSMA6P4-RP11-120E13.1*	n.98300581G>C	None	Low	-
13	98300584	98300585	*PSMA6P4-RP11-120E13.1*	n.98300585A>C	None	Low	-
13	108527191	108527192	*FAM155A-LIG4*	n.108527192C>T	rs547032144	Low	0.89
13	108542600	108542601	*FAM155A-LIG4*	n.108542601G>A	rs149383683	Low	0.29
13	108559560	108559561	*FAM155A-LIG4*	n.108559561G>A	rs541712696	Low	-
13	108857507	108857508	*LIG4*	c.*3372_*3373insTTT	rs146739883	Low	-
13	108915581	108915582	*TNFSF13B*	n.78-6866A>G	rs117976973	Low	0.50
13	109053517	109053518	*TNFSF13B-HCFC2P1*	n.109053519_109053524delACACAC	rs143572234	Low	-
13	109076930	109076931	*TNFSF13B-HCFC2P1*	n.109076931_109076932insTCTCTCTC	None	Low	-
13	109154792	109154793	*HCFC2P1-MYO16*	n.109154793G>A	rs187868150	Low	0.40
13	109325175	109325176	*MYO16*	c.226+6679T>C	rs72664976	Low	0.89
13	109453319	109453320	*MYO16*	c.676-5700dupA	rs377390365	Low	0.80
13	109595133	109595134	*MYO16*	c.1860-14902T>G	rs118168489	Low	0.89
13	109646663	109646664	*MYO16*	c.2376+1886_2376+1887dupCA	rs146565717	Low	-
13	109835672	109835673	*MYO16*	c.5349+3692A>C	rs187349074	Low	0.70
13	109863191	109863192	*MYO16*	c.*4008G>A	None	Low	-
13	110001293	110001294	*LINC01067-LINC00399*	n.110001294A>G	rs147705218	Low	0.40
13	110183896	110183898	*LINC00399-LINC00676*	n.110183898delT	rs544892286	Low	-
13	110320676	110320680	*LINC00399-LINC00676*	n.110320678_110320680delTTC	rs147586219	Low	-
13	110350350	110350351	*LINC00399-LINC00676*	n.110350351T>A	rs182466123	Low	0.80
13	110492036	110492047	*IRS2-RN7SKP10*	n.110492038_110492047delCATAATATAA	None	Low	-
**13**	**110826878**	**110826879**	***COL4A1***	**c.3326-7dupT**	**rs532261610**	**Medium**	**0.37**
13	110942287	110942288	*COL4A1*	c.84+17003G>A	rs187157903	Low	0.60
13	111189067	111189068	*RAB20*	c.173-12524A>C	None	Low	-
13	111997038	111997039	*TEX29*	c.*569C>T	None	Low	-
13	112203312	112203313	*TEX29-RP11-65D24.2*	n.112203313A>G	None	Low	-
13	112313508	112313509	*RP11-65D24.2*	c.170-11236C>T	rs551876124	Low	0.15
13	112605700	112605701	*AL136302.1-SNORD44*	n.112605701G>T	rs529136683	Low	0.14
13	112798120	112798121	*LINC00403-LINC01070*	n.112798121G>A	rs565231257	Low	0.41

Table S3. Continued

Chr	Start	End	Gene	Variant	dbSNP	Impact	MAF (%)
13	112840752	112840753	*LINC00403-LINC01070*	n.112840753_112840754insT	rs562370447	Low	-
13	112883767	112883769	*LINC01070-LINC01043*	n.112883769delA	rs369608209	Low	-
13	113276405	113276406	*TUBGCP3-C13orf35*	n.113276406G>C	None	Low	-
13	113281868	113281869	*TUBGCP3-C13orf35*	n.113281869_113281870insTGTG	None	Low	-
13	113335353	113335354	*C13orf35*	c.259+1402G>A	None	Low	-
**13**	**113534962**	**113534963**	***ATP11A***	**c.*11G>A**	**None**	**Medium**	**-**
13	113699222	113699223	*MCF2L*	c.450-362T>G	rs145185774	Low	0.85
13	113756650	113756651	*AL137002.1*	c.-43G>A	None	Low	-
13	113801835	113801836	*F10*	c.865+26C>T	rs183118165	Low	0.35
13	114073963	114073964	*ADPRHL1*	c.*3173C>T	rs147978889	Low	0.99
13	114075847	114075848	*ADPRHL1*	c.*1289T>C	rs112596428	Low	0.99
13	114075907	114075908	*ADPRHL1*	c.*1229G>A	rs111450368	Low	0.99
13	114075927	114075928	*ADPRHL1*	c.*1209A>G	rs186651486	Low	0.99
13	114076021	114076022	*ADPRHL1*	c.*1115T>A	rs112353951	Low	0.99
13	114076640	114076641	*ADPRHL1*	c.*496G>C	rs144952998	Low	0.99
13	114088197	114088198	*ADPRHL1*	c.380-16T>G	rs369286904	Low	0.70
13	114088198	114088199	*ADPRHL1*	c.380-17C>A	rs373227737	Low	0.71
13	114172890	114172891	*TMCO3*	c.1226-2040G>A	rs138992456	Low	0.50
13	114454632	114454633	*LINC00552-TMEM255B*	n.114454633C>T	rs142234274	Low	0.50
13	114481059	114481060	*TMEM255B*	c.252+8927G>A	rs146119780	Low	0.30
13	114638627	114638628	*LINC00565-RASA3*	n.114638628C>T	rs113366916	Low	0.40

Table S4. *In silico* predictions for *ATP11A*, chr13:113534962G>A

Allele	Reference	Alternate	Difference
**MaxENT**	6.43	1.43	5
**Human Splice Finder**	70.8	41.85	28.95
**Maximum Dependence Decomposition Model**	10.08	3.98	6.1
**First-order Markov Model**	5.09	1.41	3.68
**Weight Matrix Model**	7.2	4.03	3.17

Table S5. *In silico ATP11A* wildtype vs. mutant NNPLICE splice site predictions

Wildtype	*ATP11A* g.190616G>A	Sequence
**0.97**	**0.61**	**acgctaaGTaactgt**[1]
0.98	0.98	gttcacgGTgagaaa
**1.00**	**1.00**	**gtcccagGTaagtaa**[2]
0.66	0.66	caggcagGTgtgagt
0.41	0.41	cttgctgGTcagacc
0.81	0.81	ggtaccgGTatggcg
0.69	0.69	gacgaggGTgtgtcc
0.49	0.49	gatggtaGTgagtgc
0.51	0.51	tctgcagGTtcatcc

[1]Donor splice site that is documented in NCBI and Ensembl. [2]Most probable splice site, due to *ATP11A* g.190616G>A.
